# Atypical Metatarsal Fractures: Report of Five Clinical Cases

**DOI:** 10.1055/s-0043-1770906

**Published:** 2024-04-19

**Authors:** José Wanderley Vasconcelos, Naraja Menezes de Souza, Leopoldina Milanez da Silva Leite, José Alonso Rodrigues Chaves Júnior

**Affiliations:** 1 Faculdade de Medicina, Universidade Federal do Maranhão, São Luís, MA, Brasil

**Keywords:** biphosphonates, femoral fractures, fractures, bone, metatarsus, osteoporosis

## Abstract

Atypical fractures are well elucidated when they occur in the femur and are related to the use of bisphosphonates. Prolonged therapy with this drug leads to excessive suppression of bone remodeling, which makes the bone more brittle. In general, they are caused by minimal trauma or are atraumatic. This type of fracture is also reported in other bony sites, such as the metatarsus. Some reports and studies on atypical metatarsal fractures have been published, but further investigations are required to better understand this type of fracture and establish the proper diagnosis, treatment and conduct.

The present study is a report of five cases of patients who presented metatarsal fractures during therapy with bisphosphonates. All patients were female, had osteoporosis as a preexisting disease, were taking bisphosphonates, presented fractures that were either atraumatic or caused by minimal trauma, and the imaging examination showed a transverse meta-diaphyseal fracture of the fifth metatarsal shaft with thickening of the lateral cortex, image characteristics similar to the criteria used by the American Society for Bone and Mineral Research (ASMBR) to define atypical femur fractures.

## Introduction

Atypical fractures due to prolonged use of bisphosphonates in the treatment of osteoporosis were first reported in the 2000s. 1 Those that occur in the femur are well defined by the American Society for Bone and Mineral Research (ASMBR); however, reports 2 indicate that they can also occur in other bones, such as the metatarsus.

Shane et al. 2 elucidated the criteria for atypical femur fractures: association with no or minimal trauma; prodromal symptoms; fracture line originating in the lateral cortex and substantially transverse in its orientation, which may also become oblique as it progresses medially through the femur; complete fractures extend through both corticals and may be associated with a medial tip, while incomplete fractures only involve the lateral cortical; fractures that are non-comminuted or minimally comminuted; and the presence of periosteal or endosteal thickening, which appears radiologically as a “beak” in the lateral cortex of the fracture. 2

Atypical fractures of other bones besides the femur, such as the pelvis, humerus, tibia, and metatarsus have been reported in other studies. 3 4 5 6 In the first epidemiological study on metatarsal fractures, 6 published in 2020, the authors concluded that the use of bisphosphonates is associated to an increased risk of metatarsal fractures, but not to the duration of drug use.

Reports on atypical metatarsal fractures, as well as studies that provide guidance on diagnosis and management, are scarce. The aim of the present study is to describe five cases of this type of fracture in patients with osteoporosis undergoing bisphosphonate therapy.

## Clinical Cases

The present study was approved by the institutional Ethics in Research Committee; it was included in Plataforma Brasil (CAAE- 50365521.3.0000.5086), and the informed consent form was signed by the participants.

The five clinical cases are summarized in Table 1 to elucidate the most relevant information regarding atypical metatarsal fractures.

**Table 1:** Patients and data on atypical metatarsal fractures

Patient	Age in years	Atraumatic or minimal trauma	Imaging study	Preexisting disease	Bisphosphonate used (therapy duration)
**I**	77	Atraumatic	Transverse metadiaphyseal fracture of the fifth metatarsal shaft with thickening of the lateral cortex	Osteoporosis	Risedronate sodium (> 7 years)
**II**	51	Atraumatic	Transverse metadiaphyseal fracture of the fifth metatarsal shaft with thickening of the lateral cortex	Osteoporosis	Ibandronate sodium (> 5 years)
**III**	77	Atraumatic	Transverse metadiaphyseal fracture of the fifth metatarsal shaft with thickening of the lateral cortex	Osteoporosis	Alendronate sodium (10 years)
**IV**	65	Atraumatic	Transverse metadiaphyseal fracture of the fifth metatarsal shaft with thickening of the lateral cortex	Osteoporosis	Alendronate sodium (5 years)
**V**	66	Minimal trauma	Transverse metadiaphyseal fracture of the fifth metatarsal shaft with thickening of the lateral cortex	Osteoporosis	Zoledronic acid (2 years); previous use of alendronate sodium (7 years)

**Fig. 1:**
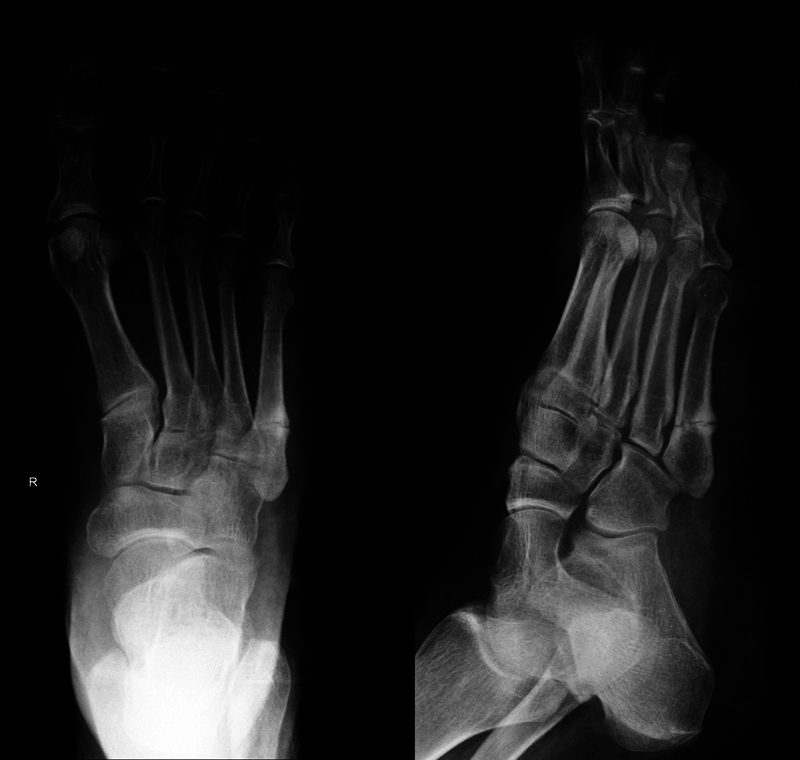
Anteroposterior and oblique radiographs of the right foot.

All patients were female, and anteroposterior and oblique radiographs revealed transverse metadiaphyseal fracture of the fifth metatarsal axis with thickening of the lateral cortex, as shown in Fig. 1 regarding patient V.

## Discussion

The patients were aged between 51 and 77 years; they all had osteoporosis as the underlying condition, and the average use of bisphosphonates to treat the disease until the moment of the fracture was of 7.2 years.

In every case, the fracture had transverse metadiaphyseal presentation of the axis of the fifth metatarsal with thickening of the lateral cortex, features similar to the criteria used by the ASMBR to define atypical femur fractures. 2

There is concern that prolonged therapy leads to excessive suppression of bone remodeling, 7 which makes bones more brittle and causes the buildup of microdamage, increasing the risk of fractures. 7

West et al. 6 concluded that the risk of metatarsal fracture among postmenopausal women taking therapeutic bisphosphonates was higher with younger age, non-Hispanic white ethnicity, and history of diabetes, rheumatoid arthritis, fracture, and treatment with glucocorticoids.

The lack of criteria to define atypical metatarsal fractures makes the diagnosis difficult, and they may be considered simple stress fractures, as occurs in the case of atypical femoral fractures, 8 or the relationships involving fractures and comorbidities and fractures and the concomitant use of other medications may not noted, as indicated in the ASMBR criteria for atypical femur fractures. 2

Therapeutic suspension may be considered for atypical fractures; however, in the case of atypical femur fractures, Brown et al. 9 indicated that it should only be performed in specific situations. Correspondingly, the management of patients with atypical metatarsal fractures may have similar indications that should be properly studied and elucidated. In the present study, we recommended that all patients discontinue the use of bisphosphonates and switch to another drug line, in addition to undergoing conservative treatment, which showed good evolution in four of the cases, one of which was still under treatment until the submission of the study.

Atypical metatarsal fractures need further studies so that the diagnosis and management are better delineated. As suggested by Unnanuntana et al. 10 regarding atypical fractures of the femur, studies on atypical metatarsal fractures that focus on bone histomorphometry and biomechanical properties should be carried out for a deeper understanding of the relationship between bisphosphonates and this type of fracture.
