# Supplementary material for: Atypical Metatarsal Fractures: Report of Five Clinical Cases
Source: Rev Bras Ortop (Sao Paulo). 2024 Apr 19;59(Suppl 1):e119–22. doi: 10.1055/s-0043-1770906 (PMC11254433; doi:10.1055/s-0043-1770906)
Supplement: Related texts

# Research article: Fraturas atípicas de metatarso: Relato de cinco casos clínicos

**Keywords:** bifosfonatos, fraturas do fêmur, fraturas ósseas, metatarso, osteoporose

## Abstract

Fraturas atípicas são bem elucidadas quando ocorrem no fêmur e tem relação com o uso de bifosfonatos. A terapia prolongada com esse medicamento leva a uma supressão excessiva do remodelamento ósseo, o que torna o osso mais quebradiço. Em geral, são fraturas atraumáticas ou causadas por trauma mínimo. Este tipo de fratura é também relatada em outros sítios ósseos, como o metatarso. Alguns relatos e estudos referentes às fraturas atípicas de metatarso já foram publicados, mas outras investigações são necessárias para que essa fraturas sejam mais bem compreendidas e recebam o devido diagnóstico, tratamento e conduta.

No presente trabalho, relatam-se cinco casos de pacientes que apresentaram fratura de metatarso durante terapia com bifosfonatos. Todos os pacientes eram do sexo feminino, tinham osteoporose como doença pré-existente, faziam uso de bifosfonatos, apresentaram fraturas atraumáticas ou causadas por trauma mínimo, e o exame de imagem revelou fratura metadiafisária transversa de eixo de quinto metatarso com espessamento do córtex lateral, características de imagem semelhantes aos critérios utilizados pela American Society for Bone and Mineral Research (ASMBR) para definir fraturas atípicas de fêmur.

## Introdução

Fraturas atípicas causadas pelo uso prolongado de bifosfonatos no tratamento da osteoporose foram inicialmente relatadas nos anos 2000. 1 As que ocorrem no fêmur são bem definidas pela American Society for Bone and Mineral Research (ASMBR), mas relatos 2 indicam que esse evento pode ocorrer também em outros ossos, como no metatarso.

Shane et al. 2 elucidaram os critérios da fratura atípica de fêmur: associação com ausência de trauma ou com trauma mínimo; sintomas prodrômicos; origem da linha de fratura na cortical lateral e substancialmente transversal em sua orientação, que pode também se tornar oblíqua à medida que progride medialmente pelo fêmur; fraturas completas se estendem por ambas as corticais e podem estar associadas a uma ponta medial, ao passo que fraturas incompletas envolvem apenas a cortical lateral; a fratura é não cominutiva ou minimamente cominutiva; e presença de espessamento periosteal ou endosteal, que se apresenta radiologicamente como um “bico” na cortical lateral da fratura. 2

Fraturas atípicas de ossos que não o fêmur, como pelve, úmero, tíbia e metatarso, foram relatadas em outros estudos. 3 4 5 6 No primeiro estudo epidemiológico sobre as fraturas do metatarso, publicado em 2020, 6 os autores concluíram que o uso de bifosfonatos está relacionado ao aumento do risco de fraturas de metatarso, mas não à duração do uso do medicamento. 6

Há poucos relatos sobre as fraturas atípicas de metatarso, bem como estudos que orientem o diagnóstico e a conduta. O objetivo deste estudo é apresentar cinco casos desse tipo de fratura em pacientes com osteoporose em terapêutica com bifosfonatos.

## Casos Clínicos

Este estudo foi aprovado pelo Comitê de Ética em Pesquisa da nossa instituição, inserido na Plataforma Brasil (CAAE- 50365521.3.0000.5086), e o formulário de consentimento livre e esclarecido foi assinado pelos participantes.

Os cinco casos clínicos foram reunidos em um quadro de maneira que são elucidadas as informações mais relevantes quanto às fraturas atípicas de metatarso ( Tabela 1 ). Todos os pacientes são do sexo feminino.

**Tabela 1:** Pacientes e informações sobre as fraturas atípicas de metatarso

Paciente	Idade em anos	Ausência de trauma ou trauma mínimo	Exame de imagem	Doença preexistente	Bifosfonato utilizado (tempo de terapia)
**I**	77	Ausência de trauma	Fratura metadiafisária transversa de eixo de quinto metatarso com espessamento do córtex lateral	Osteoporose	Risedronato de sódio (> 7 anos)
**II**	51	Ausência de trauma	Fratura metadiafisária transversa de eixo de quinto metatarso com espessamento do córtex lateral	Osteoporose	Ibandronato de sódio (> 5 anos)
**III**	77	Ausência de trauma	Fratura metadiafisária transversa de eixo de quinto metatarso com espessamento do córtex lateral	Osteoporose	Alendronato de sódio (10 anos)
**IV**	65	Ausência de trauma	Fratura metadiafisária transversa de eixo de quinto metatarso com espessamento do córtex lateral	Osteoporose	Alendronato de sódio (5 anos)
**V**	66	Trauma mínimo	Fratura metadiafisária transversa de eixo de quinto metatarso com espessamento do córtex lateral	Osteoporose	Ácido zoledrônico (2 anos); uso prévio de alendronato de sódio (7 anos)

Todas os pacientes eram do sexo feminino e, nas radiografias nas incidências anteroposterior e oblíqua, apresentavam fratura metadiafisária transversa de eixo de quinto metatarso com espessamento do córtex lateral, como mostra a Fig. 1 , referente à paciente V.

**Fig. 1:** Radiografias do pé direito nas incidências anteroposterior e oblíqua.

## Discussão

As pacientes tinham idades entre 51 e 77 anos; todas apresentavam osteoporose como doença subjacente, e a média de tempo de uso de bifosfonatos no tratamento da doença até o momento da fratura foi de 7,2 anos.

Em todos os casos, a fratura tinha apresentação metadiafisária transversa de eixo de quinto metatarso com espessamento do córtex lateral, características semelhantes aos critérios utilizados pela ASMBR para definir fraturas atípicas de fêmur. 2

Existe a preocupação de que a terapia prolongada leva a uma supressão excessiva do remodelamento ósseo, 7 o que torna os ossos mais quebradiços e causa o acúmulo de microdanos, elevando o risco de fraturas. 7

West et al. 6 concluíram que o risco de fratura de metatarso entre mulheres na pós-menopausa que faziam uso terapêutico de bifosfonatos é maior no caso de mulheres mais jovens, de etnia branca não hispânica, e com histórico de diabetes, artrite reumatoide, fratura, e tratamento com glicocorticoides.

A inexistência de critérios para definir fraturas atípicas de metatarso dificulta o diagnóstico, e elas podem ser consideradas simples fraturas por estresse, como ocorre no caso das fraturas femurais atípicas, 8 ou ainda não serem notadas as relações das fraturas com comorbidades ou com o uso concomitante de outros medicamentos, conforme apontam os critérios da ASMBR para fraturas atípicas de fêmur. 2

A suspensão terapêutica pode ser considerada para fraturas atípicas; porém, no caso de fraturas atípicas de fêmur, Brown et al. 9 indicaram que ela só deve ser feita em situações específicas. Analogamente, a conduta para os pacientes com fratura atípica de metatarso talvez tenha indicações semelhantes, que deveriam ser propriamente estudadas e elucidadas. Neste trabalho, recomendou-se a todas as pacientes a suspensão do uso dos bifosfonatos e a troca por outra linha medicamentosa, além da realização do tratamento conservador, com o qual se observou boa evolução em quatro dos casos, sendo que um ainda estava em tratamento quando da submissão deste trabalho.

As fraturas atípicas de metatarso precisam de mais estudos para que o diagnóstico e a conduta sejam mais bem delineados. Assim como sugere o trabalho de Unnanuntana et al. 10 sobre fraturas atípicas de fêmur, estudos sobre fraturas atípicas de metatarso centrados na histomorfometria óssea e nas propriedades biomecânicas devem ser feitos para que se compreendam melhor as relações entre bifosfonatos e este tipo de fratura.
